# Diseases with health disparities as drivers of COVID‐19 outcome

**DOI:** 10.1111/jcmm.15599

**Published:** 2020-08-20

**Authors:** John T. Moore, William Pilkington, Deepak Kumar

**Affiliations:** ^1^ Julius L. Chambers Biomedical/Biotechnology Research Institute (JLC‐BBRI) North Carolina Central University Durham NC USA; ^2^ HOPE Program JLC‐BBRI North Carolina Research Campus (NCRC) Kannapolis NC USA

**Keywords:** COVID‐19, health disparities, risk factors

## Abstract

The COVID‐19 pandemic has forced our society to come face to face with complex issues that were once theoretical but are now being played out in real time. As data from the pandemic accumulates, it is clear that COVID‐19 is impacting some parts of society more than others. Unfortunately, there is an almost complete overlap between COVID‐19 risk factors and conditions that are already represented as health disparities, such as hypertension, diabetes, heart disease, lung disease and immune disorders. In this review, we discuss our current understanding of the physiological and pathophysiological pathways that link these diseases to COVID‐19 outcome. An increased awareness of the factors underlying this issue, both societal and medical, is needed to understand the long‐term implications and possible solutions needed going forward.

## 1 INTRODUCTION

In late 2019, a novel coronavirus, SARS‐CoV‐2, originated in Wuhan, China, the capital of Hubei Province. The virus rapidly spread from Wuhan and within 3 months affected at least 180 countries and regions around the world. As of 12 May 2020, the World Health Organization reports that 4.1 million cases of COVID‐19 have been officially confirmed and have resulted in 283 153 deaths.^1^ In the United States, the outbreak was first reported in the state of Washington on 21 January 2020, although more recent test results indicate that introduction of the virus to the United States could have occurred weeks earlier.^2^ On 29 February 2020, the first confirmed death was reported, also in the state of Washington. Within 2 months, COVID‐19 spread to most states with 1 342 594 confirmed cases and 80 820 deaths as of 12 May 2020.^3^ The most devastating cluster associated with the outbreak continues in New York City where 35 per cent of the total COVID‐19 deaths have occurred.^4^ This pandemic has overwhelmed surge capacity in all sectors of our economy and has revealed our lack of basic preparedness for such an event.

Because the COVID‐19 pandemic is still unfolding, there is much that is unknown about the virus. For example, what is the true infection rate for COVID‐19, and can a person contract the virus again after having been once infected? Do survivors have any residual morbidities associated with the infection? The established news organizations and social media have been providing almost minute by minute updates on the virus, but many so‐called facts are contradictory, and many turn out to be false. We are just beginning to see the emergence of scientific publications that are attempting to analyse the magnitude and impact of the pandemic, but the global scientific community is still playing catch‐up with this surprising and deadly virus. COVID‐19 has changed any feelings of complacency regarding emerging viral pathogens in just the span of a few months. This virus has exhibited some surprising characteristics that have baffled both physicians and public health research scientists, not the least of these is ‘happy hypoxia’ which refers to COVID‐19 patients whose oxygen levels fall to dangerous levels without them showing any signs of distress. The virus not only attacks the lungs, but also has been widely associated with heart, liver, and kidney problems as well as fatal blood clotting. Yet, some people who contract COVID‐19 experience very few symptoms and little discomfort. So, why does this virus make some people seriously ill while others are only marginally affected? Also, why is it affecting many more men than women and more older people than younger people? Why are obese people, persons with diabetes, and those with compromised immune systems more likely to die from the virus? And now news comes that a recent mutation of the virus (Spike D6114G) has produced a much more contagious strain than the original.^5^

Recent hospital reports confirm that COVID‐19 is mostly impacting older persons, and those with underlying chronic diseases such as hypertension, diabetes, heart disease, lung disease and immune disorders.^6^ The hospitalization rate among patients identified through COVID‐NET during the most recent 4‐week period was 4.6 per 100 000 population.^7^ Rates were highest (13.8) among adults aged ≥ 65 years. Among 178 (12%) adult patients with data on underlying conditions as of 30 March 2020, 89.3% had one or more underlying conditions; the most common were hypertension (49.7%), obesity (48.3%), chronic lung disease (34.6%), diabetes mellitus (28.3%) and cardiovascular disease (27.8%). These findings suggest that older adults have elevated rates of COVID‐19–associated hospitalization and most persons hospitalized with COVID‐19 have underlying medical conditions.

Unfortunately, there is an almost complete overlap between COVID‐19 risk factors and health disparities that disproportionately affect minority populations. An increased awareness of this problem is needed to understand the long‐term implications and the underlying factors. For example, environmental issues such as air and water quality are more prevalent in poor and minority communities and contribute to many of the COVID‐19 risk factors. Living in lower income densely populated metropolitan areas, lack of health insurance and limited access to health care all likely to contribute to higher rates of infection and adverse outcome due to COVID‐19.

**FIGURE 1:**
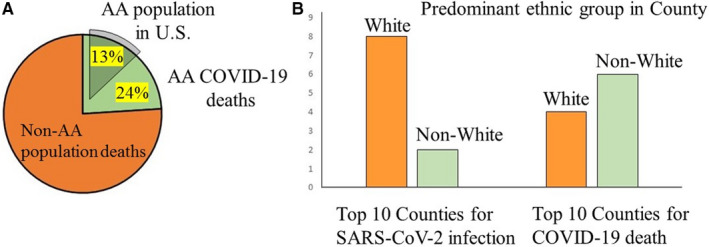
Date from The COVID‐19 Racial Data Tracker (June 2020) showing (A) Percentage of the US population that is African American (AA) vs the percentage of COVID‐19 associated deaths in AA vs the non‐AA population (compiled from cases where ethnicity is known), and (B) predominant ethic group in top 10 countries in US for confirmed SARS‐CoV‐2 infections vs COVID‐19 deaths. https://covidtracking.com/race

The COVID‐19 pandemic presents a stark reminder of the important role of social determinants of health in minority communities. Early findings that have examined COVID‐19 demographics show that minority populations are bearing a disproportionate number of COVID‐19 cases and deaths irrespective of geographic region.^8^, ^9^ The COVID‐19 racial tracker (https://covidtracking.com/race) shows clear racial disparities at every level, from the general US population, to individual counties (Figure 1). Of 131 predominantly black counties in the United States, the infection rate is 138/100 000 and the death rate is 6/100 000,^10^ an infection rate that is more than threefold higher than that in predominantly white counties. Moreover, the death rate for predominantly black counties is sixfold higher than in predominantly white counties.^8^ In Chicago, more than 50% of COVID‐19 cases and nearly 70% of COVID‐19 deaths involve black individuals, although blacks make up only 30% of the population with the deaths concentrated mostly in just 5 neighbourhoods that are predominantly African American.^11^ In New York City, close examination of a current map of COVID‐19 cases clearly shows that minority neighbourhoods are the most affected neighbourhoods in the city. If New York City has become the epicentre, this disproportionate burden is validated again in underrepresented minorities, especially blacks and now Hispanics, who have accounted for 28% and 34% of deaths, respectively (population representation: 22% and 29%, respectively).^12^ If New York City were a country, it would rank fourth in total COVID‐19 cases in the world.

African Americans in rural communities appear to face an even more challenging situation.^13^, ^14^ As of 6 April 2020, more than two‐thirds of rural US counties have been affected,^15^ and although all population groups have been affected, racial/ethnic minorities have borne the brunt of the pandemic, especially African Americans.^16^ In Louisiana, 72% of deaths related to COVID‐19 are African Americans, who comprise only 32% of the population.^17^ Similar disproportionately high death rates in African American communities are being reported in other states like Illinois, Wisconsin, Michigan and North Carolina.^10^ In this review, we discuss how COVID‐19 risk factors may interact with viral mechanisms to contribute to the observed health disparities.

## 2 ARE THERE ETHNIC FACTORS ASSOCIATED WITH SARS‐COV‐2 INFECTIONS?

SARS‐CoV‐2 belongs to the SARS family of viruses and is a positive‐strand RNA with a particle size of 60‐100 nm.^18^ It binds to the angiotensin‐converting enzyme 2 (ACE2) protein on recipient cells via its spike (S) protein.^19^ As a second step of viral entry, SARS‐CoV‐2 requires priming of the S protein by a type 2 transmembrane serine protease, TMPRSS2.^19^ TMPRSS2 is also responsible for the entry of other respiratory viruses such as influenza H1N1 and is thus being pursued as a therapeutic target.^19^ TMPRSS2 is androgen regulated which has prompted interest in a possible role of TMPRSS2 in gender differences in the pandemic. TMPRSS2 is also overexpressed in prostate cancer and is part of a prevalent oncogene fusion (TMPRSS2‐ERG)^20^ suggesting a potential link with severe outcome in males. TMPRSS2‐ERG fusions are more dominant in Caucasians when compared with African Americans or Asians but the relevance with respect to COVID‐19 is yet to be explored.

FURIN, a proprotein convertase subtilisin/kexin enzyme, is another protease that has been proposed to be involved in the entry of SARS‐CoV‐2.^21^ FURIN‐like cleavage recognition sites are present in SARS‐Co‐V2 but not in SARS‐Co‐V^22^ suggesting a unique entry and pathogenesis mode for the new virus. FURIN is known to modulate sodium‐electrolyte balance and, like the renin‐angiotensin‐aldosterone system (RAAS), plays an important role in regulating blood pressure and is dysregulated in individuals with hypertension. FURIN variants are known to be associated with elevated systolic BP (SBP) and diastolic BP (DBP).^23^, ^24^ Cilhoroz et al^24^ also demonstrated in a small number of participants that association of FURIN variants in Post‐Exercise Hypotension (PEH) is dependent upon intensity of exercise and race.

## 3 CYTOKINE RELEASE SYNDROME

Cytokines are mediators of inflammatory response and play important roles in the immunopathology of viral infection. A rapid and well‐coordinated innate immune response is the first line of defence against viral infection. However, dysregulated and excessive immune responses may cause damage to the body. Cytokine release syndrome (CRS) is a systemic inflammatory response caused by infection, some drugs and other factors, characterized by a sharp increase in the level of many pro‐inflammatory cytokines. These so‐called inflammatory or cytokine storms have been observed in viral infections where excessive or uncontrolled levels of cytokines activate additional immune cells, resulting in hyperinflammation and localized tissue destruction and broader pathological consequences including death. CRS is one of the factors contributing to the high mortality rates in COVID‐19.^25^

Clinically, COVID‐19 infection can be divided into 2 phases. During an initial non‐severe stage, a specific adaptive immune response can eliminate the virus and prevent disease progression. But if this does not occur, the disease may progress to a severe stage where lung inflammation becomes a significant cause of mortality.^26^ Therefore, good general and immune health may not be a predictor of recovery for patients who have advanced to the severe stage and once severe lung damage occurs, the clinical strategy may shift to suppression of inflammation. High levels of IL‐1B, IL‐2R, IL‐6, IL‐10, IFN‐γ, IP‐10, MCP‐1, granulocyte colony‐stimulating factor, macrophage inflammatory protein‐1A and TNF‐α have been detected in patients with COVID‐19. Higher levels of IL‐2R, IL‐6, IL‐10, TNF‐alpha and high LDH levels have been shown to be associated with severe COVID‐19 symptoms.^27^ A strong correlation has been reported between IL‐2R and IL‐6 levels and severity of the disease in critically ill patients.^28^ IL‐6 has many effects that may increase vascular leakage, increased permeability in endothelial cells, induce tissue factor expression on the cell surface of monocytes and ultimately trigger a coagulation cascade, ultimately leading to multiorgan injury.^29^ Thus, blocking some of these cytokines (most notably, IL‐6) has become a strategy for treatment.^30^ It will be an important question to determine the extent to which progression to severe stages of COVID‐19 where CRS becomes an issue contributes to the higher mortality rates in minority populations.

## 4 RISK FACTORS AND UNDERLYING CONDITIONS FOR ADVERSE outcome

Unfortunately, virtually all the underlying health conditions most strongly associated with negative outcome from COVID‐19^31^ are disproportionately higher in minority groups and those in challenging socioeconomic conditions. Thus, health disparity issues are now being compounded by the fact that individuals that were already disproportionately affected by many common chronic diseases will also disproportionately suffer both the short‐term (acute disease/hospitalization) and long‐term morbidity and mortality associated with the pandemic. A closer examination of these underlying conditions and their relationship to coronavirus is necessary to gain a complete understanding of the factors driving health disparities in the COVID‐19 pandemic.

## 5 HYPERTENSION

While high blood pressure affects all segments of the population, it is much more prevalent among African American men and women. The prevalence of hypertension in African Americans in the United States is among the highest in the world at than 40 percent.^32^ The results of a pooled analyses from current scientific literature suggest that hypertension may be associated with an up to 2.5‐fold higher risk of severe and fatal COVID‐19, especially among older individuals.^33^

There have been many recent reports concerning a possible connection between COVID‐19 disease and hypertension treatments. This is because many hypertensive patients are treated with RAAS blockers which target molecular pathways that include ACE2, the receptor that facilitates coronavirus entry into cells. The two most common classes of drugs used in this regard are angiotensin‐converting enzyme (ACE, a homolog of ACE2) inhibitors (ACEi) and angiotensin receptor blockers (ARBs). The notion has been popularized that treatment with renin‐angiotensin system inhibitors might increase the risk of acquiring the disease or developing a severe and fatal infection. The speculation was partly driven by murine studies that showed that these drugs may increase the expression of ACE2 in tissues such as lung. However, there are conflicting data from other studies demonstrating that these drugs have minimal effect on ACE2 levels (for review, see Clerkin et al^34^). As no human clinical studies to date have directly addressed this question, virtually all major medical advice groups have recommended against stopping ACEi, ARBs or other related drugs during the pandemic. The question, although, will undoubtedly be aggressively pursued as the more patient data from the pandemic become available. Further analysis is recommended because African Americans use of antihypertensive drugs is disproportionately higher.^34^

## 6 CARDIOVASCULAR DISEASE

The burden of cardiovascular disease in the African American community is high and is a primary cause of the disparity in life expectancy between African Americans and whites.^35^, ^36^ Among blacks age 20 and older, 46% of men and 48% of women have cardiovascular disease.

COVID‐19 increases morbidity and mortality in patients with pre‐existing underlying cardiovascular conditions such as coronary artery disease, heart failure, cardiomyopathy, and history of stroke and heart attack, most notably for those with severe disease (detailed statistics on cardiovascular disease and COVID‐19 are available).^6^, ^34^, ^36^ In the SARS epidemic, where more retrospective data are available, pre‐existing cardiovascular disease increased the risk of death 12‐fold.^37^, ^38^ The mechanisms driving these associations in COVID‐19 remain unclear and potential explanations include cardiovascular disease being more prevalent in those with advanced age, a functionally impaired immune system, elevated levels of ACE2, or a predisposition to acquire COVID‐19 for those with cardiovascular disease.^34^ Adding to the problem is that infection with SARS‐CoV‐2 can induce new myocardial injury and dysfunction on top of pre‐existing conditions. The SARS‐CoV‐2 entry protein (ACE2) is expressed not only in the lung, but also in other tissues such as the heart, and some studies suggest direct uptake of virus into these tissues.^39^ Myocardial injury is commonly associated with fatal outcome of COVID‐19. In an analysis of data from a study of hospitalized patients in Wuhan, patients with underlying cardiovascular disease were more likely to exhibit elevated Troponin T levels (TnT, a marker of myocardial injury) compared with patients without pre‐existing cardiovascular disease.^40^ In addition to the heart and lung, ACE2 is expressed in the intestinal epithelium, vascular endothelium and kidneys, providing a mechanism for the multiorgan dysfunction that can be seen with SARS‐CoV‐2 infection.^39^, ^41^

## 7 DIABETES

Type 2 diabetes (T2D) prevalence in the United States is significantly higher in African Americans vs whites. African American adults are 60 per cent more likely than white adults to have been diagnosed with diabetes. In addition, African Americans are twice as likely as non‐Hispanic whites to die from diabetes.^42^

COVID‐19 patients without other comorbidities other than diabetes are at higher risk of severe pneumonia, release of tissue injury‐related enzymes, excessive inflammation responses and an hypercoagulation state in blood associated with dysregulation of glucose metabolism.^43^ Thus, diabetes represents another morbidity risk factor that predicts rapid progression and poor prognosis in COVID‐19.

A variety of cytokines are significantly higher in patients with diabetes compared to those without diabetes. In diabetic patients with COVID‐19, serum levels of inflammation‐related biomarkers such as IL‐6, C‐reactive protein, serum ferritin, coagulation index and D‐dimer were significantly higher, suggesting that patients with diabetes may be more susceptible to an inflammatory/cytokine storm eventually leading to rapid deterioration of COVID‐19.^44^ IL‐6, which is already increased in conditions of chronic inflammation, may play a more deleterious role in COVID‐19 infection.^44^

## 8 CHRONIC RESPIRATORY DISEASE

A disproportionate burden of chronic respiratory diseases (mainly chronic obstructive pulmonary disease, COPD) occurs in people of low socioeconomic status due to differences in health behaviours, social factors and environmental exposures.^45^ Tobacco use, occupations with exposure to inhalant toxins, and indoor biomass fuel exposure are more common in low socioeconomic status populations.^45^

Epidemiological COVID‐19 data from Wuhan China showed that the older patients, and especially those with COPD, were at high risk of the involvement of the severe and critical type of COVID‐19.^46^ A meta‐analysis demonstrated that COPD is associated with a significant, over fivefold increased risk, of severe COVID‐19 disease.^47^ Another meta‐analysis addressed smoking and found that COPD and ongoing smoking history attribute to a worse progression and outcome of COVID‐19.^48^ Among asthma patients, male gender ,African American race, and history of diabetes mellitus were associated with higher expression of ACE2 and the protease TMPRSS2.^49^

Proteases and antiproteases secreted from the respiratory epithelium are involved in respiratory homeostasis, and changes in the protease/antiprotease balance can contribute to the development of COPD. Significantly, an altered protease/antiprotease balance, in favour of increased protease activity, is associated with increased susceptibility to respiratory viral infections such as influenza virus and with increased activation and replication of SARS‐CoV.^50^ It will be important to understand the role that a disrupted respiratory protease/antiprotease balance plays in COPD and SARS‐CoV‐2 infection.

## 9 LIVER DISEASE

Disparities exist for multiple liver diseases, including viral hepatitis, non‐alcoholic fatty liver disease (NAFLD) and hepatocellular carcinoma, with NAFLD accounting for the greatest number of affected individuals.^51^ In the United States, Hispanics are the most disproportionately affected ethnic group with NAFLD, with an estimated 45% with fatty liver vs 25% in the general population.^52^

In a study from the Wuhan outbreak, it was found that the majority of COVID‐19 patients with persistent liver injury also had NAFLD and high BMI.^53^ Post‐mortem liver biopsy of a COVID‐19 patient suggested that liver injury in COVID‐19 infection is likely immune‐mediated rather than due to direct cytopathic damage as described in other viral respiratory diseases. Patients with NAFLD also had a higher risk of progression to severe COVID‐19. With NAFLD increasing global prevalence, this may suggest a large proportion of our population could be developing an increased risk of severe COVID‐19.

Given the limited studies on liver disease and COVID‐19, it still remains unknown whether patients with pre‐existing fatty liver disease are more susceptible to COVID‐19 or whether the severity of the underlying liver disease a prognostic factor of ultimate severity of COVID‐19 disease.^54^ With regard to hepatocellular carcinoma, and other liver diseases which are less prevalent, additional clinical data are needed for a full assessment of the impact of these risk factors on COVID‐19 disease.

## 10 AUTOIMMUNE DISORDERS

Autoimmune diseases affect over 22 million Americans and are mainly represented by systemic lupus erythematosus (SLE) and rheumatoid arthritis (RA). Despite recent advances in treatment options, there remain measurable health disparities in these autoimmune disease outcome. Both SLE and RA disproportionately affect women, racial and ethnic minorities, the poor, and those lacking medical insurance and education.^55^, ^56^

Patients with a compromised immune system, and patients that are being treated with immunosuppressive drugs or autoimmune disease for cancer therapy, would be expected to be more susceptible to a severe disease course in COVID‐19.^57^ In SLE studies, it has been found that there is hypomethylation of the ACE2 gene and resulting overexpression of ACE2 in the disease. The oxidative stress induced by viral infections appears to exacerbate the DNA methylation defect in SLE, possibly resulting in further ACE2 hypomethylation and enhanced viral infection. In addition, demethylation of interferon‐regulated genes, such as NFκB, and key cytokine genes might exacerbate the immune response to SARS‐CoV‐2 and increase the likelihood of a cytokine storm.^57^

In addition to factors related to susceptibility and severity to COVID‐19 disease, modification of immunosuppressive strategies in autoimmune disease treatment in SLE and RA are being evaluated. In individuals with RA, infection risk is increased compared to the general population because of an overall impairment of the immune system typical of autoimmune diseases combined with the immunosuppressive effect generated by corticosteroids and other immunosuppressive drugs. Post‐infection, the effect of immunosuppressive drugs on disease outcome is not clear. Given that hyperactivation of the immune system may be a component of the pathology, such as during cytokine storms, some clinical studies are assessing specific anti‐rheumatic and other immunosuppressive drugs as potential treatment options for the management of COVID‐19.^58^, ^59^

## 11 EMERGING DATA AND DIFFERENTIAL outcome

Approximately 80%‐85% of patients infected with SARS‐CoV‐2 experience mild or no symptoms, while the remainder develops severe disease. As the COVID‐19 pandemic continues, more information will become available from which to decipher why certain populations are at greater risk. We have described many of the factors that are contributing to creating the observed health disparities, but root causes that unify these seemingly disparate conditions may yet be discovered. As one example, recent data gathered from African American and homeless populations suggest that vitamin D insufficiency (VDI) may be an underlying driver of COVID‐19 severity^60^ as VDI was found to be highly correlated with severe COVID‐19 in patients. This is intriguing as severe COVID‐19 disease and VDI share associations including hypertension, obesity, male gender, advanced age and immune dysfunction, as well as the fact that VDI is more common among African Americans due to differences in skin colour.^61^ Moreover, vitamin D levels are positively associated with cardiovascular respiratory fitness and negatively associated with different measures of adiposity in African American men and women.^62^ Thus, studies concerning differential response to the virus may provide new answers and avenues to ameliorate disease outcome.

## 12 THE SOCIAL DETERMINANTS OF HEALTH AND COVID‐19

In addition to the multitude of disease risk factors described above, social determinants of health also need to be examined as they profoundly impact COVID19‐related health disparities. Despite social isolation measures being implemented in most western nations, the incidence of COVID‐19 continues to rise disproportionately in minority communities in every country. Minority and low‐income communities are least likely to have jobs capable of remote working, more likely deemed as essential workers and more likely to live in high‐density areas in large metropolitan areas due to affordability, all of which intensifies their chances of getting infected. Further, minorities and low‐income community members are typically the last to volunteer for testing or take extra measures for testing due to the possibility of lost wages, stigma and mistrust. Also, implicit bias among healthcare workers (in the United States, only six per cent of physicians are African American) results in substandard care for minorities and prevents them from being proactive for testing or timely treatment.

## 13 SUMMARY

The COVID‐19 pandemic highlights many of the same issues regarding health disparity as the HIV pandemic. As with COVID‐19, African Americans are disproportionately affected by HIV and AIDS (African Americans make up 13% of the United States population but account for approximately 42% of new HIV diagnoses). The health disparity similarities of these two biologically different viruses show that many of the driving factors for both include many sociopolitical factors. Health disparities in COVID‐19 may end up being even more dramatic given that COVID‐19 is associated with many risk factors as described above that will magnify the disparity. The disparity in COVID‐19 needs to be addressed quickly as the most likely treatments for the disease, vaccine or antiviral drugs are just beginning human trials. Regardless, as has been observed in other health disparities, breakthroughs in drug discovery and medical treatments do not solve the disparity issues unless the socioeconomic issues are addressed in parallel.

## CONFLICT OF INTEREST

The authors confirm that there are no conflicts of interest.

## AUTHOR CONTRIBUTIONS

**John T. Moore:** Writing – review and editing (equal). **William Pilkington**: Writing – review and editing (equal). **Deepak Kumar:** Conceptualization (supporting); writing – original draft (supporting); writing – review and editing (equal).

## Data Availability

Data sharing is not applicable to this article as no new data were created or analysed in this study.
